# Supplementary material for: MetaRibo-Seq measures translation in microbiomes
Source: Nat Commun. 2020 Jun 29;11:3268. doi: 10.1038/s41467-020-17081-z (PMC7324362; doi:10.1038/s41467-020-17081-z)
Supplement: Supplementary file 10 — Supplementary Data 7 [file 41467_2020_17081_MOESM10_ESM.zip › File2/Confidence_High_Taxonomy/402333_out.krona.html]

Javascript must be enabled to view this page.

members
magnitude
magnitudeUnassigned
count
unassigned
taxon
rank

402333\_out

56

superkingdom
2
56

phylum
56
976

class
56
200643

171549
56
order

45
815
family

45
816
genus

SRS011084\_contig\_number\_13075SRS015694\_contig\_number\_2471SRS023583\_contig\_number\_15116SRS048981\_contig\_number\_8059SRS062654\_contig\_number\_contig-100\_476.155436SRS075021\_contig\_number\_8401SRS146812\_contig\_number\_contig-100\_535.321927
7

4
817
species

SRS015890\_contig\_number\_8205SRS018984\_contig\_number\_5806SRS023715\_contig\_number\_27749SRS024492\_contig\_number\_17717

species

SRS018049\_contig\_number\_contig-100\_448.24620SRS052078\_contig\_number\_1723SRS101268\_contig\_number\_1074
3
357276

5
457395

SRS015579\_contig\_number\_22628SRS019286\_contig\_number\_contig-100\_161.49294SRS023346\_contig\_number\_1484SRS057717\_contig\_number\_6120SRS140645\_contig\_number\_contig-100\_44.25841
species

1
46506
species

SRS043768\_contig\_number\_32048


SRS147271\_contig\_number\_29799
species
1
162156

species

SRS077127\_contig\_number\_16670
1
371601


SRS021484\_contig\_number\_contig-100\_1170.160164
species
1
329854


SRS013521\_contig\_number\_13704SRS013638\_contig\_number\_2901SRS015794\_contig\_number\_13041SRS045645\_contig\_number\_31186SRS049995\_contig\_number\_38939SRS078176\_contig\_number\_23166SRS101376\_contig\_number\_28358SRS893341\_contig\_number\_15937
species
8
1739298


SRS022137\_contig\_number\_21479SRS055982\_contig\_number\_19511SRS893342\_contig\_number\_8217
species
3
1907658

626929
1
species

SRS144135\_contig\_number\_33735

species

SRS148319\_contig\_number\_15800
1262747
1

species

SRS077589\_contig\_number\_3131SRS1041112\_contig\_number\_6562
818
2

1
821

SRS018836\_contig\_number\_contig-100\_112.166795
species

6
291645

SRS015431\_contig\_number\_56707SRS021219\_contig\_number\_8351SRS024435\_contig\_number\_43882SRS052027\_contig\_number\_18731SRS063985\_contig\_number\_32136SRS077194\_contig\_number\_contig-100\_224.192244
species

1853231
11
family

genus
574697
11

species

SRS014613\_contig\_number\_contig-100\_246.123946SRS016018\_contig\_number\_5713SRS016132\_contig\_number\_3932SRS017701\_contig\_number\_13753SRS019397\_contig\_number\_11541SRS064276\_contig\_number\_42407SRS097889\_contig\_number\_40624SRS1041137\_contig\_number\_31803SRS143342\_contig\_number\_21264SRS145497\_contig\_number\_11709SRS148196\_contig\_number\_22712
11
1472416
